# Use case for predictive physiological models: tactical insights about frozen Russian soldiers in Ukraine

**DOI:** 10.1080/22423982.2023.2194504

**Published:** 2023-03-29

**Authors:** Adam W. Potter, David P. Looney, Karl E. Friedl

**Affiliations:** U. S. Army Research Institute of Environmental Medicine, 10 General Greene Avenue, Natick, MA, USA

**Keywords:** biophysics, modelling, thermophysiology, hypothermia, frostbite, military operations

## Abstract

Biomathematical models quantitatively describe human physiological responses to environmental and operational stressors and have been used for planning and real-time prevention of cold injury. These same models can be applied from a military tactical perspective to gain valuable insights into the health status of opponent soldiers. This paper describes a use case for predicting physiological status of Russian soldiers invading Ukraine using open-source information. In March 2022, media outlets reported Russian soldiers in a stalled convoy invading Ukraine were at serious risk of hypothermia and predicted these soldiers would be “freezing to death” within days because of declining temperatures (down to −20°C). Using existing Army models, clothing data and open-source intelligence, modelling and analyses were conducted within hours to quantitatively assess the conditions and provide science-based predictions. These predictions projected a significant increase in risks of frostbite for exposed skin and toes and feet, with a very low (negligible) risk of hypothermia. Several days later, media outlets confirmed these predictions, reporting a steep rise in evacuations for foot frostbite injuries in these Russian forces. This demonstrated what can be done today with the existing mathematical physiology and how models traditionally focused on health risk can be used for tactical intelligence.

## Introduction

The US Army's investment in biophysics and biomedical modelling over the past half century has gained momentum with increased computing speed and modelling sophistication [1–9]. US Army Research Institute of Environmental Medicine (USARIEM) predictive physiological models have been built from years of scientific discovery and understanding of human physiolgy. These models quantify complex regulatory networks and expand coalescing methods towards a larger goal of developing a unified soldier phenome model that will eventually accurately predict individual responses to previously untested sets of conditions [10,11]. Building out and combining thermal, load carriage, hypoxia/altitude, injury risk, body composition and physical performance models also helps to identify critical research gaps and next steps for new laboratory and field data collection, where models do not meet. This enduring lane of soldier performance research is relevant and timely in the current revolution of military artificial intelligence (AI).

In this paper, we address yet another useful application of physiological models, prediction of the health and performance status of opponent forces.

Mathematical models and AI-based computational methods for predicting human thermal responses have been used for mission planning and for preventing cold stress injuries [12,13]. Computational models to account for human physiological and thermoregulatory responses along with data on the biophysical properties of clothing can be used to quantitatively model responses [14–19].

In short, predicting human responses requires inputs regarding four main elements: (1) the human, (2) their activity, (3) clothing worn and (4) the environmental conditions. Current AI methods are available that can directly empower the existing human physiological models with actionable information. For example, movement patterns and specifically speed could be obtained or estimated based on AI-based video and image analysis methods [20] and used to calculate metabolic heat production (i.e. activity component). Existing AI-based image classification methods could be used to decipher the appropriate types of clothing based on media or intelligence obtained images [21,22] and combined with, or compared to, clothing databases (e.g. clothing worn). Similarly, weather forecast information can be digitally sourced using automated AI methods [23,24] (e.g. environment).

In this paper, we applied elements of USARIEM’s Cold Weather Ensemble Decision Aid (CoWEDA) model to predict the health status of opposition forces. This USARIEM model has been previously well described and validated, but, in brief, the CoWEDA is a hybrid rational and empirical model. This model was built on the foundation of a six-cylinder rationally based representation of the human body and an empirically derived cold survival model [9]. CoWEDA provides useful estimates based on average responses expected for a group [16]. Tikuisis et al. [25–27] conducted foundational work to develop modelling methods specifically for depicting differences in human body composition. This model is critically presented in another paper in this special issue [28].

## Methods

At a minimum, modelling human thermal responses require inputs from four elements: (1) environmental conditions, (2) the human, (3) their activity level and (4) clothing properties. This analysis was based on USARIEM’s existing thermal models [14–16], open-source estimates of the environmental conditions, assumptions regarding Russian soldier characteristics and their activity and recent internet images depicting clothing ensembles of Russian soldiers and using comparable clothing values to make predictions [29].

### Environmental conditions

Current open-source weather forecasts were used to project conditions for a 10-day forecast in Kyiv, Ukraine, where estimates range for air temperature (Ta) were a low of −8°C and highest of 8°C, relative humidity (RH) from 48% to 78%, and wind velocity 10–19 km/h (Weather.com). To show a range of best and worst conditions, we modelled five environmental conditions that included two within a vehicle (−8°C and −20°C, 60% RH, 1 km/hr wind velocity) and three exposed outside conditions (5°C, −8°C and −20°C, 60% RH, 17 km/hr wind velocity).

### Clothing biophysics

Based on observational assessments of Russian cold weather clothing, there are some clothing properties with likely comparable values that can be used to make estimates [17,18]. If Russian soldiers used their complete eight-layer cold weather clothing system [30], they would be fairly well protected from extreme cold exposure. From observations alone, the equipment set seems adequate but not likely to be as effective (thermally protective, comfortable, movement efficient) as the best available systems used by some other armies, notably those of the Nordic countries which use superior natural fibres such as wool. Elements of most importance to soldier protection and effectiveness are focused on extremities (hands and feet) and areas of soft tissue (e.g. cheeks). The element less known, and more important in these analyses, are gloves and boots. Internet images of captured Russians in various states of dress indicated the use of various subsets of the Russian cold weather clothing system [31]. The Russian military may have been using their clothing system but not likely the complete sets (specifically using light gloves vs. cold weather mittens), and the boots from internet images were lightweight, not extreme cold weather boots. Public media also reported that Russian soldiers were trying to obtain Ukrainian military boots to replace their own inferior boots.

Biophysical measures of dry and evaporative resistances are generally required for interpreting and/or calculating a thermal response [7,8,12,13]. Biophysical properties used for the present modelling are shown in Table 1, where the uniforms are the same except for different gloves and boots.

**Table 1.** Biophysical clothing inputs for modelled simulations.

	Units	Head	Torso	Arm	Leg	Hands	Feet
Low Protection	clo	1.80	6.26	4.37	4.49	0.77	0.74
	i_m_	0.37	0.32	0.41	0.39	0.30	0.15
High Protection	clo	1.80	6.26	4.45	4.49	2.41	1.44
	i_m_	0.37	0.32	0.42	0.39	0.94	0.14

Note: clo represents thermal insulation interpreted from measured dry resistance; i_m_ represents vapour permeability index based on measured dry and evaporative resistances.

### Human inputs

Simple assumptions can be made of Russian soldiers in this analysis. The analysis considered a “best case” simulated individual as a healthy, relatively lean, young man (1.75 m; 83.5 kg; 24.9% body fat). More specific information regarding the impact of additional potential physiological stressors such as nutritional status, hydration, and sleep status was uncertain and would make thermal strain predictions more serious than in the best case, as each added stressor can have a significant impact on the thermoregulatory effectiveness.

### Activity level

Based on this analysis, the activity rate was considered as low (representative of sitting in a vehicle) (~116 W) and slowly moving (137 W) [32–35]. It is important to note that with the restricted ability for movement (e.g. in a vehicle such as a tank), the ability to maintain body heat (i.e. metabolic heat production) is significantly reduced.

## Results

### In vehicle

**Figure 1.**
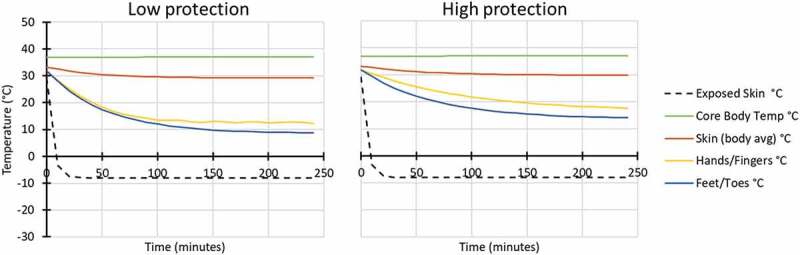
Modelled response for in vehicle: Ta −8°C, 60% RH, 1 km/hr wind velocity, while wearing low (left) and high (right) extremity protection (low protection included light gloves and all-weather boots similar to Army light leather glove and temperate weather combat boots; high protection included mittens and cold weather boots similar to Army mittens and VB boots).

**Figure 2.**
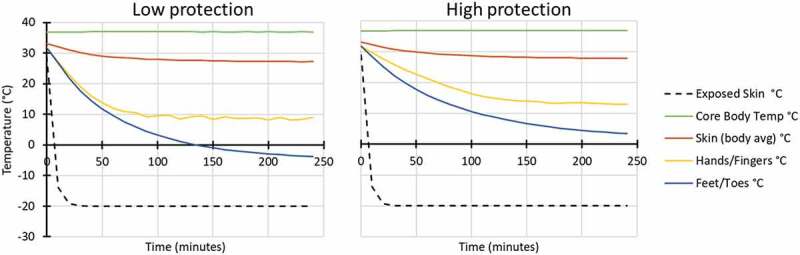
Modelled response for in vehicle: Ta −20°C, 60% RH, 1 km/hr wind velocity, while wearing low (left) and high (right) extremity protection (low protection included light gloves and all-weather boots similar to Army light leather glove and temperate weather combat boots; high protection included mittens and cold weather boots similar to Army mittens and VB boots).

Modelled conditions were conducted for four scenarios with individuals resting/sitting in a vehicle (116 W), in two air temperature conditions (Ta −8 and −20°C), with low wind penetration (60% RH and 1 km/hr wind velocity) and with both high and low hand and foot protection (Figures 1–2).

### Outside vehicle

**Figure 3.**
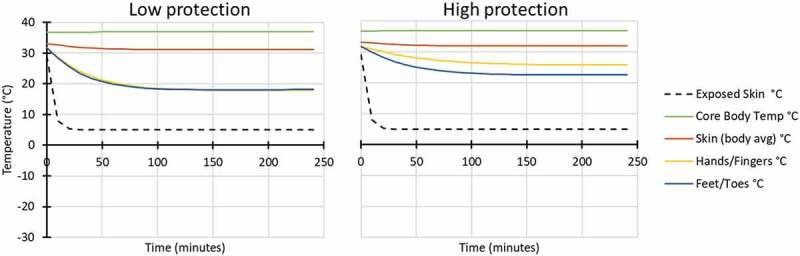
Modelled response for outside: Ta 5°C, 60% RH, 17 km/hr wind velocity, while wearing low (left) and high (right) extremity protection (low protection included light gloves and all-weather boots similar to Army light leather glove and temperate weather combat boots; high protection included mittens and cold weather boots similar to Army mittens and VB boots).

**Figure 4.**
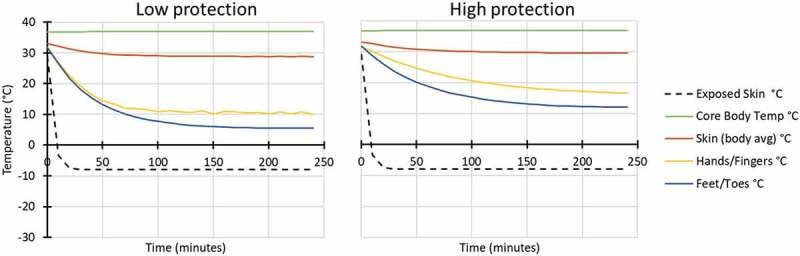
Modelled response for outside: Ta −8°C, 60% RH, 17 km/hr wind velocity, while wearing low (left) and high (right) extremity protection (low protection included light gloves and all-weather boots similar to Army light leather glove and temperate weather combat boots; high protection included mittens and cold weather boots similar to Army mittens and VB boots).

**Figure 5.**
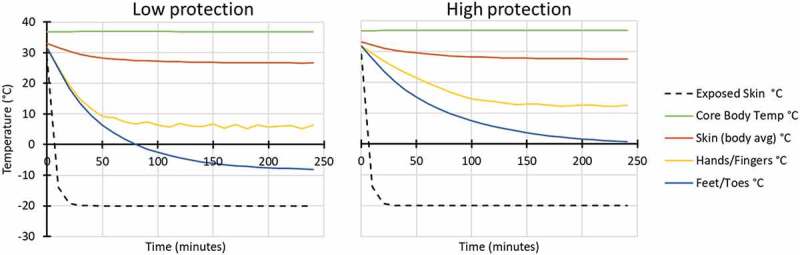
Modelled response for outside: Ta−20°C, 60% RH, 17 km/hr wind velocity, while wearing low (left) and high (right) extremity protection (low protection included light gloves and all-weather boots similar to Army light leather glove and temperate weather combat boots; high protection included mittens and cold weather boots similar to Army mittens and VB boots).

Modelling was also conducted for six conditions outside at low work rate (137 W), in three air temperature conditions (Ta 5°C, −8°C and −20°C), with exposure to wind (60% RH and 17 km/hr wind velocity) and both low and high hand and foot protection (Figures 3–5).

### Threshold points

Determining threshold points for the onset of cold-related injuries is complicated due to individual variability in responses to cold and the complexity of conditions in which they occur [13,36]. Additionally, there can be significant variation in the amount of stress that can occur to a given individual [37]. Cold responses have higher individual variability when compared to those seen in heat-related injuries. Frostbite injuries occur when skin tissue begins to freeze. Traditionally, 0°C is considered the freezing point of water, while the freezing point of skin is generally understood to be marginally lower due to things such as electrolyte levels within the tissue [36]. Additionally, observed freezing points have been seen as high as −0.6°C and as low as −4.8°C [36,38]. Hypothermia includes a broad category of cold injuries clinically described as the point at which the core body temperature drops below ~ 35°C [39].

From a modelling and simulation perspective, thresholds have been set for loss of dexterity at ~8°C, onset of pain to begin at ~5°C, and frostbite (<1°C) [13,36]. Tables 2 and 3 show the predicted times to reach general skin temperature thresholds both within the vehicle (Table 2) and outside (Table 3).

**Table 2.** Predicted times to reach thresholds within 250 minutes of exposure while in the vehicle.

Temperature (Ta)	Condition	Body Part	Numbness/Dexterity loss (8°C)	Pain (5°C)	Frostbite (<1°C)
−8°C	Low protection	Exposed skin	<10 min	<10 min	<10 min
		Hands/Fingers	-	-	-
		Feet/Toes	-	-	-
	High protection	Exposed skin	<10 min	<10 min	<10 min
		Hands/Fingers	-	-	-
		Feet/Toes	-	-	-
−20°C	Low protection	Exposed skin	<10 min	<10 min	<10 min
		Hands/Fingers	205 min	-	-
		Feet/Toes	65 min	130 min	-
	High protection	Exposed skin	<10 min	<10 min	<10 min
		Hands/Fingers	-	-	-
		Feet/Toes	130 min	185 min	-

**Table 3.** Predicted times to reach thresholds within 250 minutes of exposure while outside.

Temperature (Ta)	Condition	Body Part	Numbness/Dexterity loss (8°C)	Pain (5°C)	Frostbite (<1°C)
5°C	Low protection	Exposed skin	<10 min	<10 min	-
		Hands/Fingers	-	-	-
		Feet/Toes	-	-	-
	High protection	Exposed skin	<10 min	40 min	-
		Hands/Fingers	-	-	-
		Feet/Toes	-	-	-
−8°C	Low protection	Exposed skin	<10 min	<10 min	<10 min
		Hands/Fingers	-	-	-
		Feet/Toes	95 min	-	-
	High protection	Exposed skin	<10 min	<10 min	<10 min
		Hands/Fingers	-	-	-
		Feet/Toes	-	-	-
−20°C	Low protection	Exposed skin	<10 min	<10 min	<10 min
		Hands/Fingers	65 min	-	-
		Feet/Toes	45 min	55 min	75 min
	High protection	Exposed skin	<10 min	<10 min	<10 min
		Hands/Fingers	-	-	-
		Feet/Toes	95 min	130 min	220 min

Table 2 shows that individuals remaining in the vehicles in these conditions are at risk of frostbite to exposed skin in both the −8°C and −20°C conditions (within 10 minutes of exposure). At −8°C condition, individuals are at low risk of dexterity loss, pain or frostbite of any covered extremities with both low and higher thermal protective clothing (gloves and boots) (>250 minutes).

In the vehicle at −20°C conditions in the lower thermal protection settings, individuals are at increased risk of numbness (~65 minutes) and the onset of pain (~130 minutes) for feet and toes and increased risk of loss of dexterity of the hands and fingers (~205 minutes). In these same conditions with higher insulation protection, individuals were still predicted to be at an increased risk of numbness (~130 minutes) and onset of pain (~185 minutes) for the feet and toes.

Table 3 shows that individuals outside in 5°C conditions are at low risk of frostbite to covered areas and even for exposed skin (>250 minutes). However, there is a risk of numbness to the exposed skin in both low and high total body thermal protection (<10 minutes) and an increased risk of an onset of pain for both low (<10 minutes) and high thermal protection (~40 minutes). There is an increased risk of numbness, pain and frostbite to exposed skin in both the −8 and −20°C conditions (all within 10 minutes of exposure).

While outside at −8°C, individuals are at an increased risk of numbness to the feet and toes (~95 minutes) in lower thermal protection conditions; otherwise, there is low risk of numbness, dexterity loss, pain or frostbite with both low and higher thermal-protective clothing (gloves and boots) (>250 minutes).

Exposed to the −20°C conditions, individuals are at risk of numbness, onset of pain and frostbite of the feet and toes in both low (~45, ~55 and~75 minutes) and higher (~95, ~130 and ~220 minutes) thermal clothing conditions. There is still an increased risk of loss of dexterity to the hands (~65 minutes) in low thermal protection conditions.

## Discussion

We made predictions about cold injury risks to Russian soldiers in their current situation as described by media sources and on the basis of what we could establish regarding the environmental conditions, the soldiers, their activity levels and their clothing. For this analysis, we used USARIEM’s existing thermal models, along with open-source estimates of the environmental conditions, made assumptions regarding the ‘typical’ Russian soldier and their activity and based on some observations used comparable clothing values to make predictions. Based on these best estimates, the soldiers in this convoy were not likely to become hypothermia casualties but were likely to suffer freezing cold injuries to their feet or any exposed skin (e.g. face), especially if temperatures dipped as low as the media forecast (not the weather forecasted) of −20°C. After the fact, the temperature did not dip that low. However, following our initial results, media outlets began reporting that frostbite had become a significant issue with these Russian soldiers [40,41]

Better predictions could be made if the available artificial intelligence (AI) tools were expanded with new data and rationally based algorithms. In this respect, high priorities would be to quantify the effects of fatigue and chronic underfeeding on cold response, established qualitatively by USARIEM in a series of studies with Winter Ranger students following four 1994 hypothermia deaths in training at Fort Benning and to include cold-wet effects [42]. Another priority would be to expand the measured clothing biophysics database to include characteristics and scientifically obtained values from Russian (and/or other peer or near-peer) military uniforms. Comparison to traditional clothing (involving animal furs and different layering and wear concepts) from cold dwelling natives such as the Inuits and Sami should also be part of the database as a gold standard referent to meet or exceed with modern clothing systems. Prediction of non-freezing cold injury such as trench foot is another research gap area, where conditions leading to these conditions are known but inadequately quantified or modelled [43,44].

The AI-based physiological prediction demonstrates what can be done today with existing mathematical physiology. These AI-prediction capabilities compress the information response timeline from weeks to hours. Automation methods could be envisioned based on a collection of AI- and other existing mathematical modelling methods to quickly retrieve information for use in this type of analyses, rather than requiring a human to step through each method one-at-a-time. This capability is based on 70 years of Army efforts to systematically expand environmental physiological predictions. Initially, this required urgent seat-of-the-pants human studies. When the Army wanted to send heat acclimated soldiers from Fort Knox into the Aleutian Islands in 1943, the Armored Medical Research Laboratory (AMRL) was asked to provide clothing requirement predictions [45]. Uniformed physiologists rushed to conduct experiments with two soldiers in various clothing sets walking on treadmills in a cold room to develop clothing guidance within a few weeks, and they provided the best available advice with limited understanding of the variability and other factors. Similar human studies produced data to design guidance for cooling and ventilation requirements in Sherman tanks. However, these early data formed the start of mathematical physiology that continues at USARIEM today to provide ever-improving predictions of soldier health and performance outcomes in extreme conditions, using AI tools that can predict complex physiological interactions in minute-hours.

### Disclaimer

The opinions or assertions contained herein are the private views of the authors and are not to be construed as official or as reflecting the views of the Army or the Department of Defense. Citations of commercial organisations and trade names in this report do not constitute an official Department of the Army endorsement or approval of the products or services of these organisations.
